# Editorial: Emerging roles of extracellular vesicles in immunomodulation during host-pathogen interactions

**DOI:** 10.3389/fimmu.2022.958179

**Published:** 2022-07-22

**Authors:** Susanta Kar, Albert Descoteaux, Budhaditya Mukherjee, Leonardo Nimrichter

**Affiliations:** ^1^ Division of Molecular Microbiology and Immunology, Council of Scientific & Industrial Research (CSIR)-Central Drug Research Institute, Lucknow, India; ^2^ Institut National de la Recherche Scientifique, Université du Québec, Laval, QC, Canada; ^3^ School of Medical Science and Technology, Indian Institute of Technology, Kharagpur, India; ^4^ Department of Microbiology Geral, The Paulo de Goes Institute of Microbiology, Federal University of Rio de Janeiro, Rio de Janeiro, Brazil

**Keywords:** host-pathogen, exosomes, immunomodulation, membrane vesicle (MV), cytokines

Exosomes or extracellular vesicles (EVs) are intercellular mediators of host-pathogen interaction that play a critical role in the dissemination of pathogen and host-derived molecules during infection (1). EVs contain microbial components that trigger innate immune responses by increasing the production of proinflammatory effector molecules like reactive oxygen species (ROS) or stimulating cytokine and chemokine release (2). EVs also contain pathogen-derived antigens and pathogen associated molecular patterns that induce cross-priming to activate antigen-specific CD4^+^ and CD8^+^ T cell expansion. In contrast, exosomes from infected cells also inhibit cytokine production by T cells (3). Thus, microbial and host components can spread beyond infected cells through exosomes to either activate or suppress immune responses, collectively influencing the outcome of infection (4). The focus of this Research Topic is to shed light on recent findings that illustrate the role of EVs in regulating immunostimulatory/immunosuppressive responses that are either essential for host immunity or for pathogen-mediated immune escape.

Exosomes derived from *Mycobacterium tuberculosis*-infected macrophages activate various immune-related phenomena, from inflammatory responses to antigen presentation (5). In a study by Zhang et al., authors conduct proteomics profiling of serum exosomes isolated from normal individuals and patients of active tuberculosis (ATB). Subsequent pathway and functional analysis helps decipher the functions of differentially expressed proteins, suggesting that proteins are selectively packaged inside exosomes during various physiological conditions. These differentially expressed proteins include major histocompatibility complex (MHC) class I, CD36 (cluster of differentiation 36), and lipopolysaccharide (LPS) binding protein (LBP), all of which are routinely associated with ATB infection and may serve as potential biomarkers for the diagnosis of *Mycobacterium tuberculosis* infection.

The immunoregulatory mechanisms underpinning hepatic dysfunction during the erythrocytic stage in *falciparum* malaria are poorly understood. Using murine model of PbANKA infection, a study by Wu et al., identify a previously unknown role of galectin-receptor interactions in liver inflammatory responses. They observe that blockade of galectin-receptor interaction with alpha (α)-lactose is associated with inhibition of interferon (IFN)- IFN-α, IFN-γ and TREM-1 (triggering receptor expressed on myeloid cells) expression, leading to increased inflammatory cell infiltration, hepatocytic damage, and apoptosis. These findings further consolidate evidence for the involvement of IFN-α and IFN-γ in aggravating inflammation-induced hepatic injury, while providing insights into the regulation of innate immunity that could lead to the development of strategies that target the galectin-receptor interaction to suppress or attenuate malarial liver pathologies.

The importance of bacteria membrane vesicles (MVs) in disease pathogenesis has been well-established by evidence from multiple studies. In a review by Villageliu and Samuelson, authors summarize the functional diversity of MVs derived from Gram-positive and Gram-negative bacteria, including *Salmonella enterica*, *Klebsiella pneumonia*, *Neisseria gonorrhoeae*, and *Bacillus subtilis*. They discuss several instances of how MVs can exacerbate disease, propagate antibiotic resistance and even participate in metabolic process that benefit host health. The authors discuss examples of how antibiotic usage triggers the production of MVs by microbes like *Pseudomonas aeruginosa* and *Staphylococcus aureus*, owing to their ability to carry antibiotic-degrading enzymes like β-lactamases. Interestingly, MV release following antibiotic treatment induces biofilm formation in *S. aureus*, thereby limiting treatment efficacy (6). Collectively, these studies give us reason to believe that MVs contribute to bacterial pathogenicity, suggesting that they might be clinically relevant therapeutic targets.

Emerging evidence suggests that manipulation of host-microbe cross-talk occurs *via* MV-derived cargoes including DNA (7), RNA (8), and signalling peptides (9). The authors provide substantial literature references to highlight how bacteria-derived vesicles facilitate cross-kingdom communication and influence pathogenesis of type II diabetes by interfering with insulin signalling and glucose homeostasis (10). In this section, the authors discuss how certain microbes like *P. aeruginosa, S. aureus, Escherichia coli* and *Helicobacter pylori* secrete virulent MVs LPS, invasins, bacterial toxins, lipoglycans and proteases and compromise host defence signalling (11).

The authors further reference studies detailing how MV cargos derived from *Enterococcus faecium* and *Lactobacillus rhamnosus* carry unique metabolites and immunomodulatory molecules like indole and dopamine, which makes them attractive candidates for drug and vaccine delivery. Besides their pathogenic potential, the membrane vesicle research discussed also features works highlighting their beneficial aspects, by citing examples of how MVs rewire the host immune system, downregulate inflammation, and mediate cross-kingdom signalling.

In a review article by Zhou et al. the authors discuss the role of host EVs (HEVs) as key players of host protection by neutralizing pathogenic bacteria toxins, promoting cytokine release, and mediating antigen presentation and immune-mediate killing. In contrast, EVs released by bacteria (BEVs) may act as toxin delivery systems to exert virulence, detoxify reactive oxygen species (ROS) derived from immune cells, and induce protection from LPS-induced inflammatory responses, thereby contributing to immune escape. The authors further summarize recent studies highlighting the ramifications of EV-mediated signalling in respiratory, gastrointestinal, and urinary systems. In this regard, they discuss several examples of how EVs released from *P. aeruginosa, H. pylori*, and *Salmonella typhimurium*, stimulate acute inflammatory responses to cause airway hyperresponsiveness, shed toxins to promote artheroschlerotic plaque formation, and in some cases, attenuate tissue injury by regulating the delicate balance between regulatory T cells and Th17 cells. Collectively, by summarizing recent advances that highlight EVs as carriers of chemotherapeutic drugs like doxorubicin, and gene therapy drugs like siRNA, this review underscores the translational potential of EVs in disease diagnosis, treatment, and prevention.

## Concluding remarks

Our understanding of EVs in the context of host-pathogen interactions is still in its infancy. Nevertheless, the studies curated under this Research Topic will allow for a greater understanding of virulence mechanisms, immune responses, and overall significance of host-secreted EVs in modulating immune cell crosstalk. Clearly, more work is needed to delineate exosome function and composition during infection. Additionally, this should include defining the cell types that secrete exosomes, exosome recipient cells, and the intracellular signalling pathways impacted by exosome release. Equally critical is research into the development of methods that can be employed to block exosome production in order to evaluate disease outcome and understand whether exosome release benefits the host or is employed by the pathogen for the purpose of immune evasion.

## Author Contributions

SK, AD, BM and LD conceived and wrote the manuscript. All authors contributed to the article and approved the submitted version.

## Funding

SK’s work is supported by Council of Scientific and Industrial Research (CSIR), Department of Biotechnology (BT/PR32490/MED/29/1457), Govt. of India; Department of Science and Technology (DST, CRG/2020/002932), Govt. of India. AD’s work is supported by the Canadian Institutes of Health Research (PJT-156416). LN’s is supported by grants from the Brazilian agency Conselho Nacional de Desenvolvimento Científico e Tecnológico (CNPq) and FAPERJ.

## Conflict of Interest

The authors declare that the research was conducted in the absence of any commercial or financial relationships that could be construed as a potential conflict of interest.

## Publisher’s Note

All claims expressed in this article are solely those of the authors and do not necessarily represent those of their affiliated organizations, or those of the publisher, the editors and the reviewers. Any product that may be evaluated in this article, or claim that may be made by its manufacturer, is not guaranteed or endorsed by the publisher.
